# Health Science Research at a Regional Level: Insights From South America: Comment on "The Roles of Regional Organisations in Strengthening Health Research Systems in Africa: Activities, Gaps, and Future Perspectives"

**DOI:** 10.34172/ijhpm.2023.8076

**Published:** 2023-07-16

**Authors:** María Belén Herrero, Beatriz Nascimento Lins de Oliveira

**Affiliations:** ^1^Department of International Relations, Facultad Latinoamericana de Ciencias Sociales (FLACSO), Buenos Aires, Argentina; ^2^Consejo Nacional de Investigaciones Científicas y Técnicas (CONICET), Buenos Aires, Argentina; ^3^Centre for Social Studies, Coimbra University (CES/UC), Coimbra, Portugal

**Keywords:** Health Research, Global Health, COVID-19, Regionalism, South America, Global South

## Abstract

The article seeks to shed light on the role of regional organizations in strengthening health research systems in Africa, how they operate and how they work, as well as debts and future challenges. As can be observed also in South America, the continued strengthening of health research requires strategic thinking about the roles, comparative advantages, and capacity of regional organizations to facilitate the flourishing of health research systems. Health research is a strategic field for the transformation of socio-health inequalities on the one hand and the reduction of regional asymmetries on the other. Thus, regional organizations represent key actors in strengthening health research systems and the regional research agenda reinforces its sovereign condition in the autonomous definition of relevant topics and financing. In this process, integration mechanisms face a great challenge, as shown by the recent pandemic, not only in Africa but also in South America.

 The article “The roles of regional organizations in strengthening health research systems in Africa: activities, gaps, and future perspectives”^1^ discusses the role of international organizations in strengthening health research systems on the African continent, which emerges as an urgent topic in these times.

 The authors note that while regional cooperation in health in Africa is not new, the institutional landscape of regional cooperation for health and health research has undergone important changes, especially since the pandemic. This is interesting, since health emergencies can be an opportunity to strengthen regional organizations in support aspects of national health preparedness and response.

 In order to understand what regional organizations are doing to strengthen health research systems in Africa, the authors conducted a mapping of regional organizations involved in health sciences research and 18 interviews with key informants.

 Regarding the findings, the authors point out two factors that are fundamental and at the same time a challenge also for other regions and, in particular, for South America. One of them is that most organizations reported activities in governance and use of research, and that participation in governance was mainly focused on agenda setting and policy harmonization. The other fundamental aspect is that — according to the authors — the continuous strengthening of health research requires strategic thinking about the roles, comparative conditions and the efficacy of regional organizations to facilitate the capacity and growth of research systems in health.

 The reality presented has its specific contexts, which are quite well explained in the article and help to understand the role of public policies and international cooperation mechanisms in the field of health research. Although the scenario of the African continent is particular, some aspects relate to the reality of health research in South America, as well as the context of health cooperation. As happened in Africa, in Latin America, for decades regionalism has been influenced mainly by international trade and economic interests and, during health emergencies, by disease threats to public health. Some decades ago, health gained more interest within organizations in regional governance in both regions and due to health crises, have increased attention to the role of regional bodies in health sector planning and response, but with less attention in other aspects, such as developing research institutions, networks, or infrastructure. However, while Africa faced the COVID-19 crisis on a regional scale, Latin America, and especially South America, appeared particularly disjointed, something that also was reflected in fragmented regional cooperation.^2^

 It should be noted that, from the perspective of the Global South, health research systems must be strengthened by the construction of public policies that support national governments in preparing for and responding to emergencies and health crises, in research and in knowledge generation. This requires a political decision that allows prioritizing health research on national and regional political agendas. Although the realities of South America and Africa are different, it is essential to emphasize that, in both cases, the political decision is key.

 The authors point out that regional cooperation is a useful approach to reduce inequities in the research capacity of national health research systems. They suggest, for example, the development of a regional laboratory to develop the efficiency of countries with little or no national research infrastructure. This implies that health research cannot be solely in the hands of the private sector, since they are not committed to the public interest.

 Although the private industry invests a lot in the development of new health technologies, the COVID-19 pandemic has nevertheless shown us that without public investment in research and development, we would not have had as many safe vaccine options in such little time. The vaccines from Oxford-AstraZeneca, Sinovac and the Gamaleya Institute, as well as the case of Cuba, which developed and carried out clinical tests of 3 vaccines in national territory, are proof of the important role of public investment in strengthening national systems of health research.

 The strategic alliance between the public sector and the private sector in the field of health research is important. In any case, it must be aligned not only with private interests, but — and above all — with the needs of the population. Here, the public sector plays a key role in control, monitoring and regulation, and the regional level can strengthen the decision-making level of the public sector.

 Since the authors found that the highest performing countries, as well as those with the greatest human resources and institutional capacity, have generally benefited from substantial long-term international partnerships and collaborations, they therefore highlight that relying on international funding and alliances to develop research at the national level runs the risk of generating inequalities between countries. This point is fundamental given that these asymmetries, as well as the strong dependence on international financing, are also observed in South America. That is why it is of vital importance that countries can strengthen integration mechanisms and generate autonomous cooperation instances that tend to foster and strengthen national and regional capabilities, towards sovereign health research. Multi-stakeholder alliances are also fundamental, for the combination of resources and capacities, between state actors and non-state actors. Here, south-south cooperation and triangular south-south cooperation can be a tool in the development of projects and initiatives to strengthen regional and national health research.

 In the particular case of the South American region, the Union of South American Nations (UNASUR) and the South American Institute of Government in Health (ISAGS) had been created, in 2008 and 2011, respectively. The institute functioned as a think tank, which promoted the exchange of information and health research in different areas, such as access to medicines, disaster management, strengthening of health systems, among others.^3^ ISAGS, created with strong support from the Oswaldo Cruz Foundation — a large Brazilian science and technology research and production institution — worked actively in Rio de Janeiro between 2011 and 2019, bringing together the 12 countries of South America. With the gradual departure of eight of the 12 countries, UNASUR ended up dissolving, and so the ISAGS. It can be said that this has been a lost opportunity given that the countries gave up an important instance of production and exchange of knowledge, as well as a space for regional advocacy and international insertion in the field of global health.^4^

 Another of the authors’ interesting results — based on the interviews — is that drug and treatment policies in the African continent constitute one of the key aspects of regulatory policy, on which regional organizations focus their harmonization efforts to improve the inspection, approval and use of quality and affordable medicines. This brings us significantly closer given that one of the greatest advances on the regional agenda in South America in recent years has been in the area of medicines, through the price bank and joint purchases of high-cost medicines, which have been a great opportunity for strengthening national and regional capacities.^5,6^ Regarding the Medicines Price Bank of UNASUR, the Group on Universal Access to Medicines of UNASUR, developed a database with the prices of drugs in all the countries of the region as a negotiating strategy. This information was a very useful tool during negotiations with the pharmaceutical companies. About, the joint negotiation of prices for high-cost medicines, this initiative contributed to save millions of dollars in joint purchases of medicines for hepatitis C.^5,6^ Studies have shown that the articulation of countries through regional integration mechanisms such as UNASUR and the Southern Common Market, and with the participation of other regional instances, such as Pan-American Health Organization, led to economic savings in terms of drug purchases, with strategies of complementarity between the mechanisms, contrary to the overlapping of initiatives, such as the Joint Purchases of Medicines. Unfortunately, some of these processes were cut short due to the dissolution of UNASUR.

 Another interesting finding is that study informants perceived the greatest gap in health research when it comes to continental or interregional coordination. On this point, the importance of regional coordination mechanisms could also be of great importance in reducing this gap. During the pandemic, the absence of such mechanisms in South America can be considered as an important explanation to why there was not a concerted response.^4^

 In agreement with the authors, health is increasingly attracting the attention of regional organizations, along with more traditional aspects of regional cooperation such as trade or security, and regional organizations in the global south are considered important political forums within multilevel governance of health. However, as this study also mentions, the way in which health policy is framed and understood as an issue for regional cooperation varies between organizations and has been shown to be influenced by context-specific social, economic and political views on health policy.^7^

 The regional failure that the dissolution of UNASUR implied for South America makes us think about the importance of sustaining and strengthening the mandates of regional organizations, at least to guarantee the achievements in multilateral regional spaces, especially when having to face periods of crisis.^4^ The lack of political will of a country or group of countries has great relevance in the performance of multilateral organizations: in the end, international organizations are what the States want to make of them. With stronger mandates from member States, organizations can improve their international insertion, expand advocacy, improve governance and optimize their performance. In fact, the articulation and coordination of regional actions at the three levels — national, regional, and global — are essential.^8^ At the national level, it is necessary with a view to local capacities and, especially, strengthening health systems to meet the demand and cover the need for medical supplies and equipment, a point that has been critical in many countries in the region in times of pandemic.^4^ At the regional level, joint action is needed to articulate and foster cross-border cooperation; to guarantee the coordination of flights that transport equipment and, now, vaccines; to exchange data and promote joint mechanisms for the production and acquisition of inputs. Finally, on a global scale, coordination facilitates greater access to multilateral organizations in order to join forces to act together and negotiate as a block, understanding and defending health as a right. Joint action also has a backdrop: the possibility of reducing asymmetries, which have been evident in this pandemic crisis and which the consequences of inequitable access to vaccines will reflect more harshly.^4^ Here, health science research displays a key role. It can sum the capacities, and resources and generate knowledge to address the health needs of member states. The pandemic has demonstrated the need to develop research around new inputs, medicines, and vaccine products, as well as social research. At this point, it should be noted that regional organizations have become central actors in foreign policy, with growing relevance in the international health agenda, positioning issues on the agenda according to the needs identified by the Member States, and based on their capacities. These successive processes can come together in the elaboration of the aforementioned regional research agenda, as input for the design of public health policies, capable of resolving and responding to the needs and expectations that are typical and genuine of the region.

 Understanding then, in agreement with the authors of the article, that health research is a strategic field for the reduction of socio-health inequalities, a starting point is the identification and definition of core issues at the national level/regional, based on a diagnosis of deficiencies, asymmetries and the urgent needs of each population. Some of the elements that should be considered for this are the focus on health as a right, the participation of the main local actors (at different levels); the identification of regional needs and capacities; a health research agenda, at the regional level; regional consensus mechanisms in research; financing of the public research system; and instances of monitoring and evaluation of research in health sciences.

 A central point in this article is that while regional organizations mostly do not fund improvements to health research infrastructure, regional centers of excellence — as noted in the article — have been cited as opportunities for development of the health research infrastructure at the regional level. As some interviews point out, this is a particularly important role for regional health organizations to convene research networks that foster equity in research collaborations.

 A valuable resource in this regard is the promotion and creation of regional research networks, that is, cooperation networks between leaders in this area, in order to add resources to achieve shared goals and objectives. These networks must be constituted based on horizontal structures of co-participation and collaboration, in accordance with research strategies and action plans agreed upon by all participants, promoting the integration of all government areas and national and regional research systems involved with each problem in particular.

 In South America and within the scope of UNASUR, six structuring networks have been formed: Network of National Health Institutes, Network of Technical Health Schools, Network of Public Health Schools, Network of National Institutes of Cancer, Network of Offices of International Relations of Health, and Network of Management of risks and mitigation of disasters. With the dissolution of UNASUR, the first 4 networks — which were the most active — found other institutional paths and, after the necessary adjustments, they continued to exist and develop important work of institutional articulation in the areas of action of each one. In this way, regional organizations, especially in the current global system, can also become knowledge-generating centers to obtain, evaluate, and disseminate information on the countries’ health policies. Likewise, they can generate the necessary alliances to establish project evaluation and monitoring procedures that serve as an input for the states when evaluating the impact of their policies.

 Thus, regional organizations can become a central actor in the field of sovereign health research and in the autonomous definition of relevant topics and in the financing of an agenda (which is often subject to the “priorities” established by external funders). In this process, the integration mechanisms have a great challenge, and these studies shed light on the lessons learned, the limitations and the way forward.

## Ethical issues

 Not applicable.

## Competing interests

 Authors declare that they have no competing interests.
